# A Microneedle-Based Electrochemical Sensor with Multiplex Detection in Physiological Environment and Incorporated in a Portable Device

**DOI:** 10.1021/acsomega.5c04024

**Published:** 2025-08-05

**Authors:** Chinmay Thatte, Fiona Barry, Cláudia Ferreira, Eoin O’Keeffe, Valeria Cioce, Paul Galvin, Sofia Rodrigues Teixeira

**Affiliations:** † University College Cork, 261183 Tyndall National Institute, Lee Malting, Cork T12 R5CP, Ireland; ‡ School of Biological Sciences, 587895 Munster Technological University, Cork T12 P928, Ireland; § School of Biomedical Engineering, Politecnico di Torino, Turin 10129, Italy

## Abstract

The number of disorders linked to deficiencies in vital ions, such as calcium (Ca^2+^) and potassium (K^+^), has dramatically increased in recent years. Numerous research studies have proposed the development of a sensor for early detection; however, the bulk of these investigations include the time-consuming process of drawing blood samples for testing. To overcome these challenges, this research highlights the development of a novel portable microneedle-based device for the multiplexed detection of Ca^2+^ and K^+^, as well as pH, in artificial interstitial fluid (ISF). A passivation layer of ArCare was investigated to improve the stability, reliability, and repeatability of the microneedle sensors, enhancing their sensitivity and accuracy. Microneedle sensors were modified with Poly­(3,4-ethylenedioxythiophene) (PEDOT) and an ion-selective membrane (ISM) to reduce interference caused by other ions when tested in complex media. Successful modification of the surface with PEDOT and ISM was confirmed using electrochemical characterization, Raman Spectroscopy, and Scanning Electron Microscopy (SEM) analysis. The results demonstrated a linear response in the detection of both Ca^2+^and K^+^ ions with respect to concentration, with a high coefficient of determination (*r* ^2^ >0.9) indicating strong reliability. Additionally, tests were carried out to study the effect of pH on the detection of Ca^2+^ and K^+^, revealing a linear detection for both ions across varying pH levels. The multiparametric detection of pH and Ca^2+^ or K^+^ was carried out using an in-house built smart sensing platform known as Portable Unit Lab-on-Site Electrochemistry (PULSE). Overall, this work highlights the potential of multi-ion microneedle sensors in portable real-time health monitoring applications.

## Graphical abstract

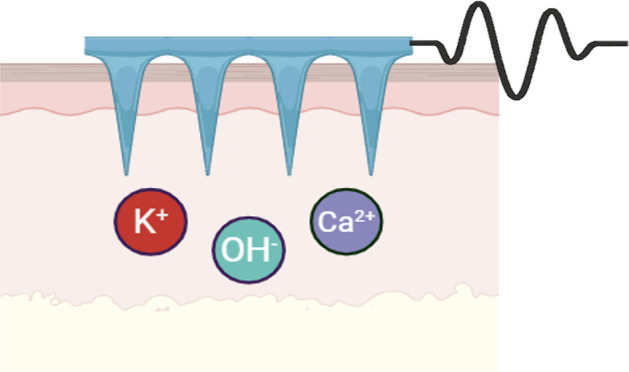

## 1 Introduction

Multiparametric electrochemical sensors offer enhanced functionality, reduced complexity, and improved efficiency compared to single-parameter sensors.^1^ Within biomedical applications, they track vital physiological markers such as glucose, lactate, pH, and temperature within body fluids, fostering continuous health surveillance and tailored medical approaches.^2^ Integrating multiple sensing components into a single platform, often utilizing cutting-edge materials and fabrication methodologies, elevates the sensors’ performance across a spectrum of parameters. Their adaptability extends to diverse domains, including environmental monitoring, healthcare provisioning, food safety, and industrial process oversight.^3^ Real-time tracking of varied parameters provides dynamic insights into biological states, enabling timely interventions. In clinical diagnostics, pharmaceutical research, and environmental analysis, multiplex biosensors play pivotal roles in optimizing resource utilization. Understanding biosensor behavior yields valuable insights into sensitivity, selectivity, detection thresholds, and response times, underpinning dependable assessments across many applications.^4^ A list of multiplexed electrochemical sensors is presented in Table 1.

**Table 1:** Examples of Multiplexed Electrochemical Sensors, Including the Biomarker, Methodology Used, and Obtained Results

biomarker	approach	results	refs
CEA and AFP	platinum nanoparticles/amino groups paired with specific metal ions and antibodies	LR: 0.05 ng mL^–1^ to 200 ng mL^–1^	^5^
		LOD: 0.002 ng mL^–1^ CEA	
bacterium detection: simultaneous detection of two common food-borne pathogens	gold/magnetic nanoparticles on a screen-printed carbon electrode. The presence of the bacterium’s protease enzymes enables rapid detection within 1 min	linear responses were obtained from 10 to 10^7^ colony-forming unit mL^–1^ for both bacteria	^6^
SARS-CoV-2, SARS-CoV-2 RNA	a four-way junction hybridization is presented; one of the four adaptor strands is modified with a redox mediator facilitating detection, using an electrochemical biosensor	LOD: 5.0 SARS-CoV-2	^7^
		LOD 6.8 μg/μL SARS-CoV-2 RNA	
multiple analytes: glucose, lactate, pH, and temperature	an array-based flexible sensor platform in polyimide was developed for the detection of multiple analytes	LOD: 26.31 μA/mM·cm^–2^ for glucose	^8^
		LOD: 1.49 μA/mM·cm^–2^ for lactate, 54 mV/pH 0.002 °C1^–^ for temperature	
carcinoembryonic antigen (CEA) and α-fetoprotein (AFP)	ITO was coated with graphene oxide for immobilization of anti-CEA and anti-AFP	LR: 0.01–300 ng/mL	^9^
		LOD: CEA is 0.650pg/m	
		LOD: AFP is 0.885pg/mL	
biosensor X	this design provides multiple immobilization areas, followed by their individual electrochemical cells. The immobilization area and electrochemical cell are strictly separate to not avoid sensitivity	LOD: 22.5 ng mL^–1^ and 28.6 ng mL^–1^ for the single analyte/sample and multiplex, respectively	^10^

Researchers are extensively working on the creation of rapid and cost-effective point-of-care (POC) diagnostic tools, investigating body fluids such as ISF, urine, and saliva as substitutes for blood. Particularly, ISF shares a similar composition with plasma, facilitating the continuous surveillance of diverse biomarkers, thereby transforming the landscape of blood-focused clinical diagnostics.^11^ Traditional methods of sampling ISF face challenges in POC diagnosis, including limited volume (1–10 μL) and filtration constraints.^11^ Microneedle technologies offer potential in transforming diagnostic protocols, bridging the gap between innovative concepts and tangible healthcare implementations. By creating microperforations in the skin, these needles bypass blood capillaries and facilitate swift healing within minutes. Given the diverse concentrations of ISF throughout the skin layers, the dermis emerged as an optimal site for microneedle-based sensing. Diagnostic apparatuses primarily employ arrays of microneedles integrated with biochemical sensors, thereby ensuring a comfortable and minimally invasive sampling process.^12^

Biosensor adoption faces ongoing drawbacks, especially with regard to stability and miniaturization. Timely disease diagnosis and monitoring hinge on continuous tracking of electrolyte levels. Hence, a mechanically resilient and multiplexed microneedle-based sensor is crucial for comprehensively detecting dynamic fluctuations, enabling the continuous monitoring of multiple electrolytes. Microneedles exist in diverse configurations—such as hydrogel-based, solid, coated, and hollow structures—each engineered to optimize specific functional parameters. Their respective advantages and application domains are detailed in Table 2.

**Table 2:** Overview of the Different Types of Microneedles and Their Uses and Limitations

microneedles	features	limitations	refs
solid	used as electrochemical biosensors with freedom of surface modification, easy to design, sturdier than other types of microneedles, and can be designed for drug delivery though skin pretreatment	limited ability for drug delivery as it relies on passive diffusion, thus requiring multiple additional steps	^13^
hollow	used for fluid extraction in ISF, drug delivery, suitable for wearable biosensors, and can be used for real-time sampling	the hollow needles are easily clogged, not easy to fabricate, and delicate if exposed to mechanical stress	^14^
coated	the example of this type of microneedle is in a controlled drug delivery via dissolvable coating, with rapid surface interaction	imitated use due to limited capacity, coating thickness can interfere with the dissolution rate	^15^
hydrogel	these are useful in electrochemical sensors for continuous detection and highly biocompatible	swelling kinetics may cause slower response, cannot adhere to mechanical force	^16^

An overview of the current state-of-the-art advancements in multiplexed microneedle-based electrochemical sensors is presented in Table 3.

**Table 3:** Overview of the State of the Art of Multiplexed Microneedle-Based Electrochemical Sensors, Including the Biomarker of Interest, Methodology Used, and Obtained Results

biomarker	approach	results	refs
phosphate, uric acid, creatinine, urea	polymer microneedle cross-linked MeHA was used for extraction of ISF through the application of mechanical strength. Four working electrodes are modified with specific enzymes or sensing reagents to achieve selective detection of respective substrates	LOD: 50–550 μM, phosphate	^17^
		LOD: 4.19 nA/μM, uric acid	
		LOD: 12.58 nA/μM, creatinine	
		LOD: 44.6 mV/decade for urea	
glucose, uric acid, and cholesterol	flexible microneedle and a multichannel portable electrochemical analyzer (MPEA) were fabricated. Needles modified with glucose oxidase (GO*x*), uricase (UO*x*), and cholesterol oxidase (ChO*x*) for simultaneous detection of glucose, uric acid, and cholesterol levels in serum	LR: glucose, 2–12 mM; uric acid, 0.1–1.2 mM; cholesterol, 1–12 mM	^18^
		LOD: glucose, 260 μM; uric acid, 4 μM; cholesterol, 440 μM, rapid response time (∼4s)	
epinephrine, dopamine, and lactate	multiplexed sensors were fabricated via assembly on a PDMS microneedle platform coated with a conductive PDMS/carbon nanotube (CNT)/cellulose nanocrystal (CNC) composite	each sensor rapidly ∼2 min LOD: epinephrine, dopamine, and lactate sensors were 0.0007 ± 0.0002 μM, 2.11 ± 0.05 nM, and 0.07 ± 0.07 mM, respectively. The pH sensor accurately responded to a pH range of 4.25–10	^19^
sodium (Na^+^) and potassium (K^+^)	microneedle-based potentiometric sensing system for multiplexed and continuous monitoring of Na^+^ and K^+^ in the skin ISFs. The potentiometric sensing system consists of a miniaturized stainless-steel hollow microneedle	LOD: 56.08 mV/decade for Na^+^	^20^
		LOD: 50.03 mV/decade for K^+^	
glucose	microneedle was initially coated with a thin layer of gold and subsequently modified to conjugate dendrimers containing a redox mediator and the catalytic bioreceptor glucose oxidase (GO*x*)	sensitivity: 0.1622 μA mm^–1^ cm^–2^; LOD: 0.66 mm	^17^
morphine and fentanyl	multimodal hollow microneedle sensor array relies on unmodified and organophosphorus hydrolase (OPH) enzyme-modified carbon paste (CP) microneedle electrodes for detection of the fentanyl and nerve agent targets, respectively	both morphine and fentanyl contribute to the oxidative current at +0.7 V when using a mixture of these drugs; however, these drugs can be separated based on the morphine peak at +0.2 V	^18^

Although ISF holds promising insights, accessing this fluid is restricted by the skin’s natural barrier.^19^ Microneedles provide a solution by effectively overcoming this barrier, allowing painless and efficient access to ISF. Characterized by their small scale, microneedles stand out as a minimally invasive approach. This method involves painless insertion into the skin, guaranteeing convenient on-body portability and the possibility of continuous biomarker monitoring.^11^

Sodium (Na^+^), Potassium (K^+^), pH, and Calcium (Ca^2+^) are key analytes traditionally evaluated in ISF due to their relevance in disease detection and association with primary diseases.^19^ Ionic deficiencies can lead to various diseases ranging from mild to life-threatening. Na^+^ is linked to fluid loss and cystic fibrosis;^20^ K^+^ is associated with arrhythmia, renal failure, sepsis, reduced gut motility, and cystic fibrosis; pH levels are related to metabolic and respiratory acidosis, sepsis, and spreading depression;^20^ and Ca^2+^ is connected to coagulopathy, osteopenia, neuromuscular excitability, and cardiovascular complications.^21^ Additionally, Ca^2+^ plays a critical role in various bodily systems, including the development of bone and teeth strength, the functioning of enzymes, and muscle contraction.^22^ Furthermore, Ca^2+^-ion fluctuation within the blood can be a sign of either hypocalcemia or hypercalcemia. Hypocalcemia is characterized by low blood calcium levels, usually less than 8.8 mg/dL.^23^ This disorder is typically associated with low vitamin D levels or issues with the parathyroid glands. In contrast, hypercalcemia is characterized by high blood Ca^2+^ levels, usually greater than 10.5 mg/dL. There are many reasons associated with high blood Ca^2+^, including hyperparathyroidism, cancer, sarcoidosis, excess vitamin D intake, certain drugs, Milk-Alkali syndrome, and Paget’s disease of the bone.^24^ The normal Ca^2+^ concentration within the human blood usually ranges from 2.1 to 2.6 mM/L.

Gang Xu et al.^25^ developed an electrochemical patch for ex vivo detection of Ca^2+^ in artificial body fluids, utilizing gold nanoparticles and a layer of graphene oxide. The working electrode (WE) was coated with 4 μL of a Ca^2+^ selective membrane, demonstrating high sensitivity when compared to ex situ samples and enabling real-time analysis using sweat. Kai Zhao et al.^26^ created a wearable sensor for Ca^2+^ detection in sweat, using a flexible polyethylene terephthalate substrate. This study observed that Ca^2+^ levels increased as the pH decreased, allowing for real-time determination. Xinhua Huang et al.^27^ designed a calcium sensor for monitoring Ca^2+^ ions within the subcutaneous tissue in the skin. This sensor was coated with a poly polystyrene sulfonate (PEDOT:PSS) solution and had PEDOT prepared on the surface of the microneedle tip, achieving a sensitivity of 21.65 mV/degree.

Recent observations from the state of the art indicated that a decline in dietary ion intake can result in a higher prevalence of ion deficiency diseases compared to previous times.^28^ These ions are essential for maintaining the electrical potential across cell membranes, nerve impulse transmission, and muscle contractions. Particularly critical for heart function, potassium aids in regulating cardiac rhythm and preventing arrhythmias. Additionally, K^+^ ions contribute to fluid and electrolyte balance in the body, supporting overall hydration and blood pressure regulation.^29^ Potassium deficiency can directly lead to hypokalemia, characterized by a drop in potassium concentration below the normal range of 3.5 to 5.0 mM/L. Conversely, if potassium levels surpass this range, the condition is termed hyperkalemia. Cardiac conduction abnormalities represent the most common and potentially consequential cardiovascular manifestations of potassium deficiency. Hypokalemia reduces the extracellular to intracellular potassium ratio, leading to the hyperpolarization of neuronal and muscular membranes. This impairs both nerve signal transmission and muscle contraction, with proximal muscles typically affected more than distal ones. In severe instances, hypokalemia can result in respiratory muscle paralysis, leading to hypoxia, hypercapnia, and potentially fatal outcomes.^29^ Building on the reviewed literature, Table 4 presents the proposed microneedle sensor, emphasizing its novel design such as integrated solid-state ion-selective layers or minimally invasive geometry and its enhanced performance in terms of sensitivity, response time, and biocompatibility. Compared to existing ion detection platforms, this sensor offers improved analytical precision, reduced sample volume requirements, and the potential for continuous in situ monitoring.

**Table 4:** Overview of the Proposed Sensor Based on the Existing Ion Detection Technologies

existing study	description	limitations	advantages of proposed sensor
hollow stainless-steel microneedle^16^	capable of detecting sodium and potassium using hollow needles with silver–silver chloride electrodes	very bulky, invasive, prone to clogging, not optimized for comfort or portability, and not easy to fabricate	the proposed solid microneedle design is minimally invasive, easy to manufacture, and highly portable, ideal for real-world, real-time monitoring
porous carbon microneedles^30^	designed for sodium detection with models for potassium using carbon needles and ion-selective membranes	not capable of multi-ion detection and no calcium detection, with limited specificity	designed for targeting clinically relevant analytes with enhanced specificity and sensitivity tested in artificial ISF
LOT-enabled polystyrene microneedles^31^	supports multi-ion detection; incorporates energy harvesting and embedded electronics	complex operation, high power consumption, large size, and not miniaturized	this sensor offers a compact, low-power design that fits in a pocket, combining simplicity with the reliability of a potentiostat
solid stainless-steel microneedle^32^	capable of detection of multiple ions using ion-selective membranes, proved much sturdier way of detection as compared to hollow microneedles	not incorporated in the portable device for practical use	designed for integration with a portable lab-on-site device for testing ions and pH anytime and anywhere with good accuracy

This work aims to develop microneedle-based sensors capable of detecting various analytes with a particular focus on potassium, calcium, and pH.

## 2 Material and Methods

### 2.1 Materials

All chemicals used were of analytical grade, and water was of ultrapure grade. Poly­(3,4-ethylenedioxythiophene) (PEDOT), Phosphate Buffered Saline (PBS), Ferrocene carboxylic acid (FcCOOH), Potassium Ionophore, Potassium tetrakis­(4-chlorophenyl) borate, Polyvinyl chloride, 2-nitrophenyloctyl ether, Tetrahydrofuran (THF), Calcium ionophore, Potassium Chloride, Calcium Chloride, Sodium Chloride, Magnesium Chloride, HEPES, Magnesium Sulfate, Sodium Phosphate, Sucrose, Hydrogen peroxide (H_2_O_2_ 0.5 mL; 30%), oxalic acid dehydrate (∼250 mg), and potassium carbonate were obtained from Sigma-Aldrich-UK. Iridium­(IV) chloride was purchased from Alfa-Aesar (Massachusetts, United States). A 1 mM FcCOOH solution was prepared in 10 mM PBS. PEDOT film was synthesized by initially preparing a 0.1 M potassium chloride solution. Subsequently, 1 mL of PEDOT is thoroughly mixed into the 0.1 M KCl solution to produce a 0.01 M PEDOT solution. The microneedles, fabricated using micromolding techniques previously established by the research group, were passivated.^33^

### 2.2 Electrochemical Measurements

Initial electrochemical measurements were conducted with an Autolab MAC90389 electrochemical workstation and Faraday cage (Metrohm, Cheshire, UK) using NOVA 2.1 software. Portability was demonstrated using an in-house built smart sensing platform known as Portable Unit Lab-on-Site (PULSE), previously reported by Ferreira et al.^34^ All measurements were carried out at room temperature using the microneedle array as the WE. For initial measurements, a silver/silver chloride (Ag/AgCl) reference electrode (RE) (Gamry, Warminster, PA 18974, USA) and a (Platinum) Pt wire (Gamry Instruments, PA, USA) as a counter reference (CE) were used. On the portable system, unpassivated gold (Au) and Pt microneedle arrays were used as the CE and RE, respectively. Additionally, for the portable system, 3D printed supports (Formlabs 3D printer with white resin, Massachusetts, USA) were produced, which held the electrodes in place and provided electrical connection to the PULSE system, as illustrated in Figure 1. A bespoke cap was used to completely seal any possible gap between the support and the array.

**Figure 1:**
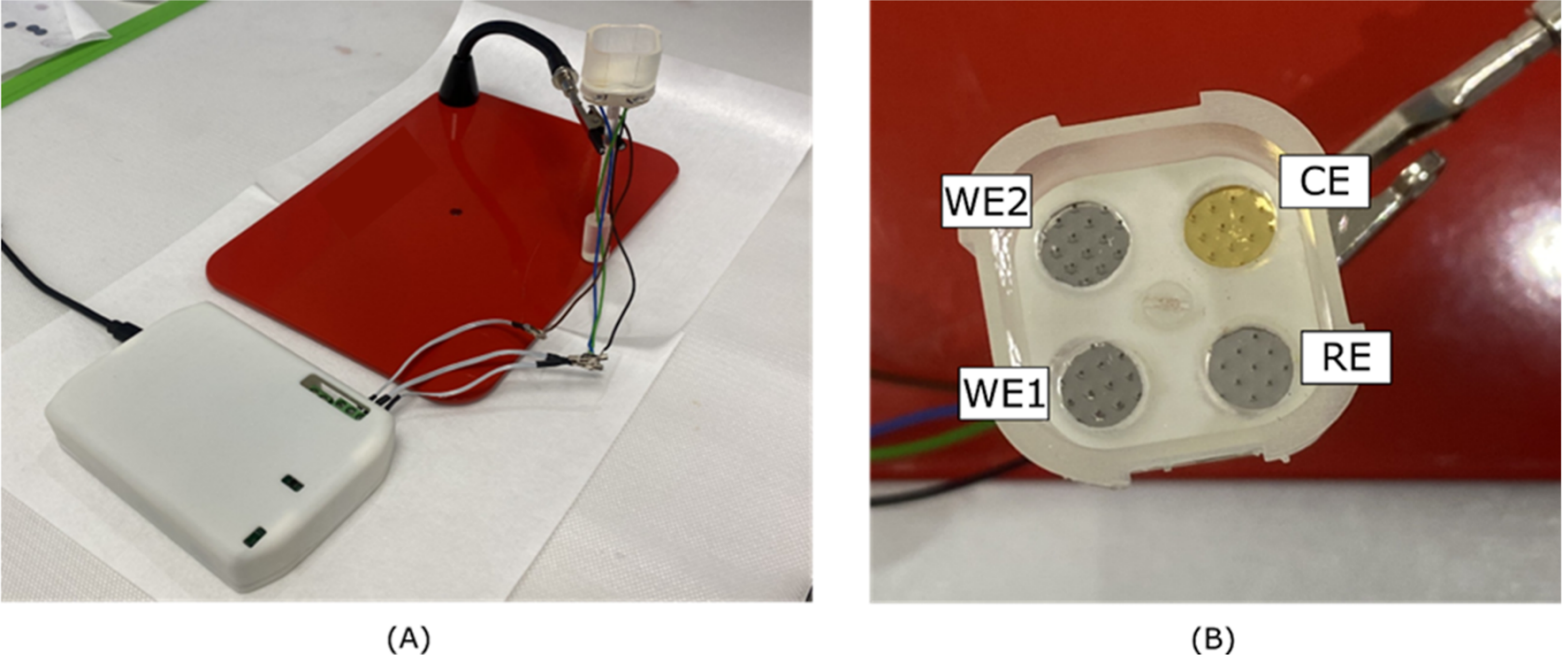
(A) Setup used with PULSE (white box) connected the microneedle’s holder and (B) top view of the microneedle’s holder, where WE1 detected K^+^ and WE2 detected Ca^2+^ (photograph courtesy of Claudia Ferreira. Copyright 2025).

Cyclic voltammetry (CV) was used for cleaning the microneedle array and characterization of the different steps for the modification. For the cleaning step, the potential was swept from −0.5 V to 0.5 V for 10 scans at 0.15 V/s, while during characterization, the potential ranged from 0 to 0.6 V with a scan rate of 0.15 V/S.

### 2.3 Passivation of Microneedles

A passivation technique was used to expose the tip of the microneedle. The adhesive film application ArCare 7759 (Adhesives Research, Limerick, Ireland) with dimensions of 152.4 mm × 9 mm was used. A Graphtec CE7000-40 (Graphtec, UK) was used to cut the ArCare film into circles with a 10.5 mm diameter. Circular holes were cut to match the size of the individual microneedles. Once cut, the film was peeled, aligned with the microneedles, and attached.

### 2.4 Electrode Modification

The electrodes were modified for the detection of different ions and pH, followed by PEDOT modification for providing ion-selective membrane attachment. Iridium oxide was used for pH measurement as a site for hydrogen and hydroxide ion exchange.

#### 2.4.1 PEDOT Modification

Chronoamperometry was used to deposit PEDOT (Poly­(3,4-ethylenedioxythiophene)) onto the exposed surface of microneedles. 1 V potential was applied for 30 s with an interval time of 0.1 s.

#### 2.4.2 Iridium Oxide (IrOx) Modification

Iridium Oxide was deposited using cyclic voltammetry. 50 cycles were implemented to get the optimal and stable deposition of the IrOx. For CV, the start potential is set at −0.7 V and upper vertex potential at a 0.7 V scan rate is set at 0.1 V/s. 50 cycles are divided in three different scans (20, 20, and 10 cycles) with a 5 s interval in between.

### 2.5 Ion-Selective Membrane for K^+^ and Ca^2+^

An ISM was employed for both the K^+^ and Ca^2+^ measurements. The ISM designed for K^+^ contained 2% K^+^ Ionophore, 0.6% potassium tetrakis­(4-chlorophenyl) borate, 32.7% Polyvinyl chloride, and 64.7% of 2-nitrophenyloctyl ether diluted in Tetrahydrofuran. For Ca^2+^, the ISM comprised 32.7% Polyvinyl chloride, 65.4% of 2-nitrophenyloctyl ether, 1.3% calcium ionophore, and 0.6% potassium tetrakis­(4-chlorophenyl) borate, also diluted in Tetrahydrofuran.

After the membrane solution was prepared, it was applied to the WE surface and allowed to dry for a minimum of 8 h at room temperature.

### 2.6 Electrochemical Analysis of K^+^, Ca^2+^, and pH

Ca^2+^ was detected in distilled water in the concentration range of 1 mM to 200 mM. Square wave voltammetry (SWV) was employed for detection purposes. Square wave measurements utilized a potential range from −0.6 V to 0.4 V, with a step size of 0.005 V, a frequency of 25 Hz, an interval time of 0.4 s, and a scan rate of 0.1 V/s. A peak response was observed at – 0.2 V; therefore, the current measured at this potential was used to construct the calibration curve. OCP (Open-Circuit Potential) measurements were taken in triplicate to detect different levels of pH, varying from 3 to 11.

### 2.7 Interference Studies

To evaluate the sensitivity of the K^+^ and Ca^2+^ sensors, the voltammetry response was evaluated in the presence of potential interference. For interference testing of calcium and potassium, 50 mM of Calcium chloride, 50 mM of sodium chloride, 25 mM of potassium chloride, and 25 mM of magnesium chloride were selected as these ions interact with the sensor’s Ion-selective membrane most when tested in ISF. These concentrations, approximately an order of magnitude higher than typical levels in ISF, were intentionally selected to evaluate the sensor performance under challenging conditions.

### 2.8 Interstitial Fluid

Based on the formulation by Bretag et al.,^35^ with slight modifications to suit specific requirements, artificial ISF was prepared by dissolving 0.0554 g of calcium chloride, 0.476 g of HEPES, 0.0522 g of potassium chloride, 0.0168 g of magnesium sulfate, 1.4236 g of sodium chloride, 0.036 g of sodium phosphate, and 0.506 g of sucrose in 200 mL of water. The pH was adjusted to 7.5. When testing, ISF was spiked with calcium chloride or potassium chloride solutions ranging from 1 mM to 200 mM concentrations.

## 3 Results and Discussion

### 3.1 Passivation Layer

To evaluate the electrical isolation provided by the passivation layer, CV was performed by using a 1 mM FcCOOH redox probe. This electrochemical characterization of the modified microneedle array surface was conducted with a three-electrode setup. In previous work, various passivation layers were developed. From all, ArCare was selected to be used in this study.^33^

ArCare passivation covered the base of the microneedle array, leaving most of the microneedles exposed. As seen in Figure 2, CV of the passivated sensor (red line) is significantly smaller than the control, i.e., nonpassivated (Blue line), proving its efficiency by covering the base. This passivation layer was found to be very reliable, providing repeatability with almost no variation. This was assessed by taking a CV for each sensor and comparing it with one another. The exposed needle provides sufficient exposure for electrochemical activity. The Arcare film covers the base, properly minimizing the interference. The exposed metal surface offered minimal resistance to the electrical current, ensuring a more direct connection between the microneedle array and the testing environment. Consequently, more current flows through the system, leading to higher currents.

**Figure 2:**
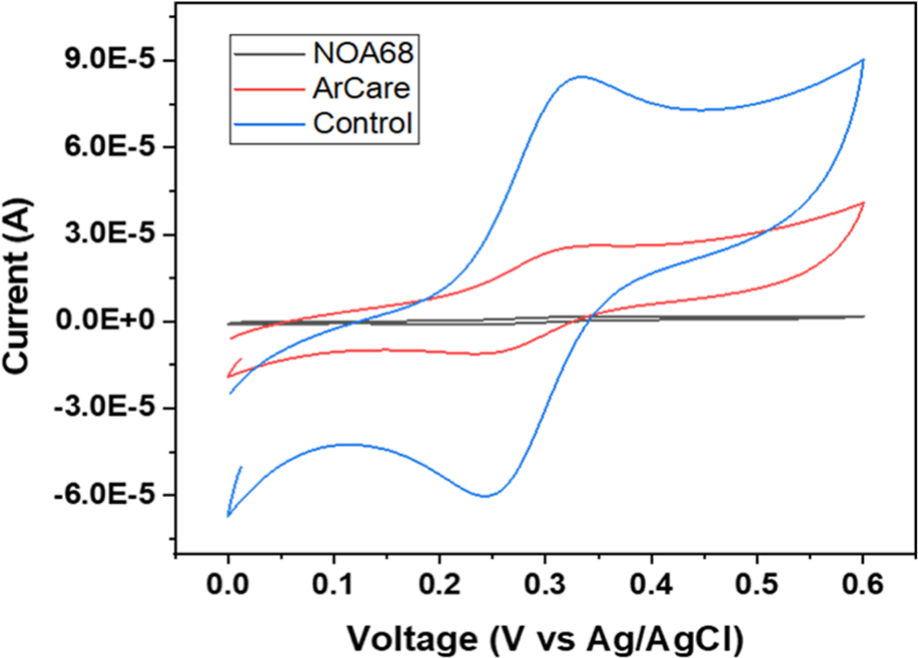
CV scans of Control, ArCare, and NOA68 in 1 mM FcCOOH in 10 mM PBS.

Another passivation layer, NOA68, was evaluated, and the tests yielded lower current values compared to ArCare (Figure 2, black line). This reduction in current is due to the limited exposed conductive surface of the microneedle array. With only the tip of the needle visible, there is less direct contact between the microneedle and the surrounding environment, resulting in lower current. All the data for this passivation study are in Supporting Information.

Figure 3 (A) displays the CV plots measured at scan rates of 10 mV/s to 150 mV/s. A linear relationship was observed between the peak oxidation and reduction currents and the square root of the scan rate (coefficient of determination (*r* ^2^) >0.97, see Figure 3 (B) for reference). These results indicated that fast electron transfer can occur at the microneedle electrode. An approximate geometric area for a bare microneedle was calculated as 4.3 mm^2^. Using the Randles–Sevcik equation, the electrochemical active area of *n* = 3 samples was determined to be 4.01 mm^2^ for ArCare passivation, which suffered a 13.6% increase after PEDOT modification to 4.56 mm^2^. Since ISM deposition provides exclusive attachment for a specific ion, the electrochemical active area was reduced to 0.23 mm^2^ for K^+^ and 0.16 mm^2^ for Ca^2+^.

**Figure 3:**
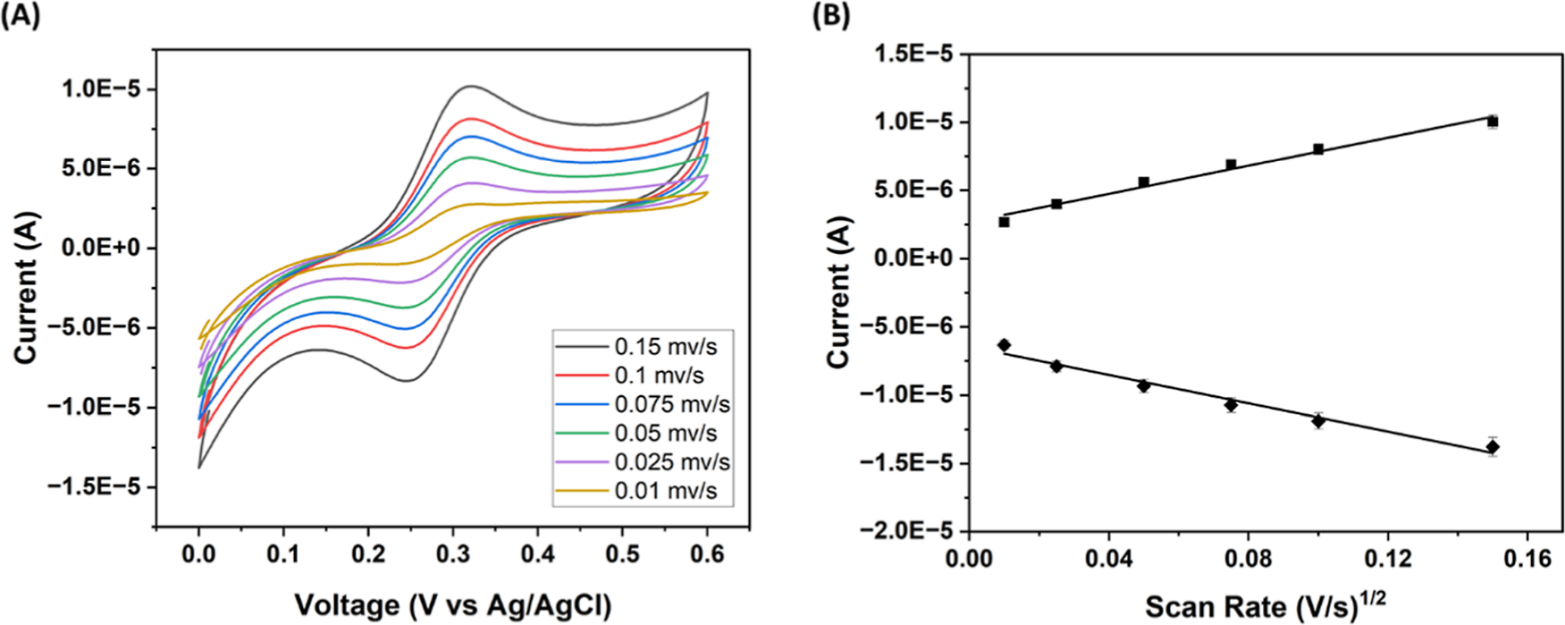
CV scans were measured on a Pt microneedle electrode at increasing scan rates (10 mV/s up to 150 mV/s). (A) ArCare passivation. (B) Corresponding plot peak current vs v^1/2^ for the different stages of the sensor.

### 3.2 Raman and SEM Characterization

Raman studies were carried out to further determine the vibration modes of the PEDOT in the wavenumber range of 500 to 2000 cm^–1^, as shown in Figure 4. The peaks at 1450 cm^–1^ can be attributed to the symmetrical stretching of C_α_ = C_β_ (in the monomer EDOT, C_α_ is the carbon atoms connecting with the sulfur atom, while C_β_ is the carbon atoms connecting with C_α_). The peaks at 1287 cm^–1^ are assigned to the C–C inter-ring stretching. The peaks at 1091 and 632 cm^–1^ are assigned to the C–O–C stretching and C–SC bending, respectively. A peak from C–C antisymmetrical stretching mode can be seen at 999 cm^–1^. The resulted spectrum is just similar to those of other results and the absence of any signal in the 650 to 680 cm^–1^ regions of the spectrum could lead to similar conclusion that the electrodeposited PEDOT chains have a highly planar structure.^36–38^

**Figure 4:**
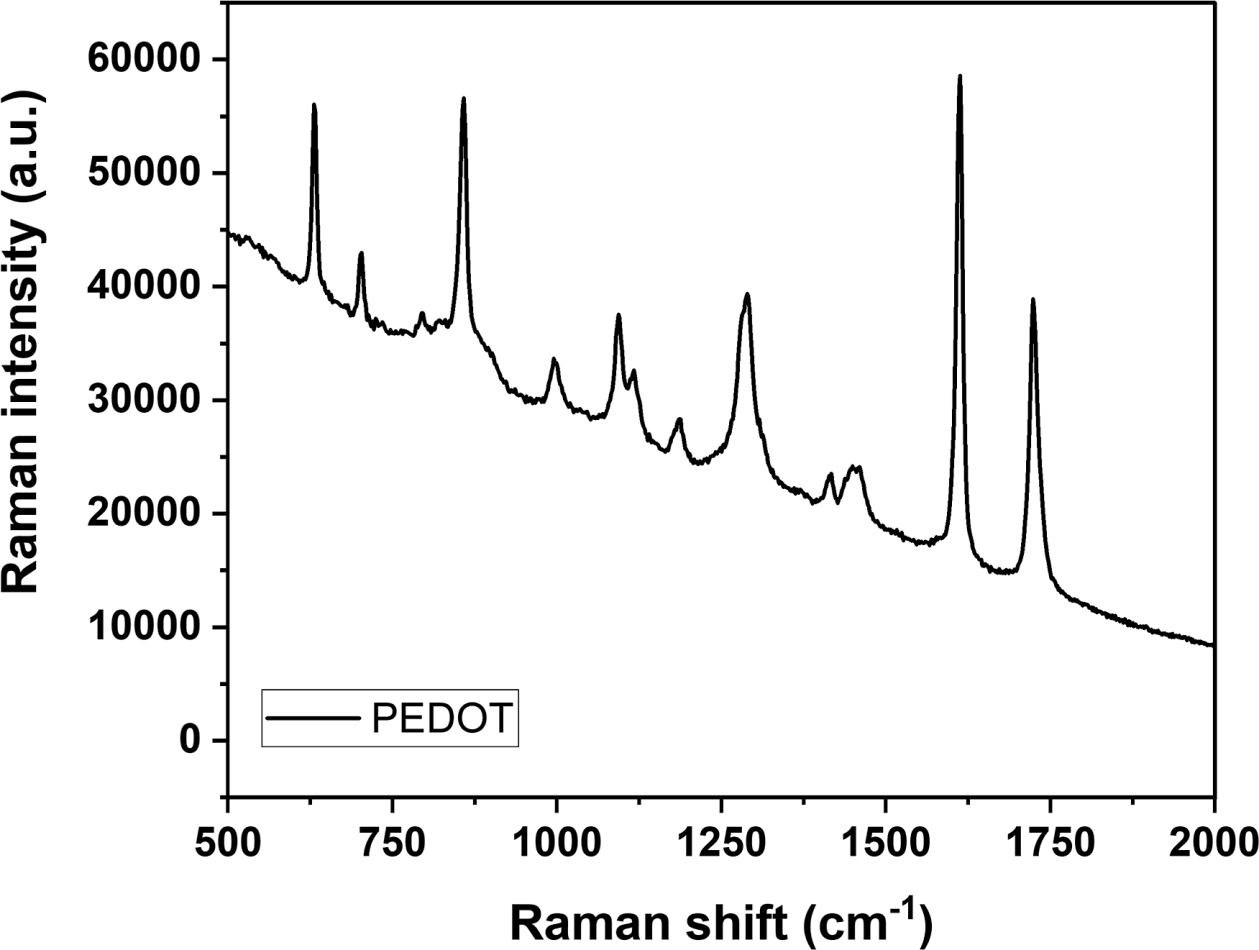
Raman spectra for PEDOT modification.

The change in the platinum microneedle surface morphology following deposition of PEDOT and, subsequently, an ISM layer can be seen in Figure 5 (A–F). PEDOT was deposited onto the platinum surface in a thin uniform layer, which can be seen in Figure 5 (D) with a magnification of 40000×. The addition of the ISM layer resulted in the array being covered in a web-like structure, highlighted by Figure 5 (E) and (F).

**Figure 5:**
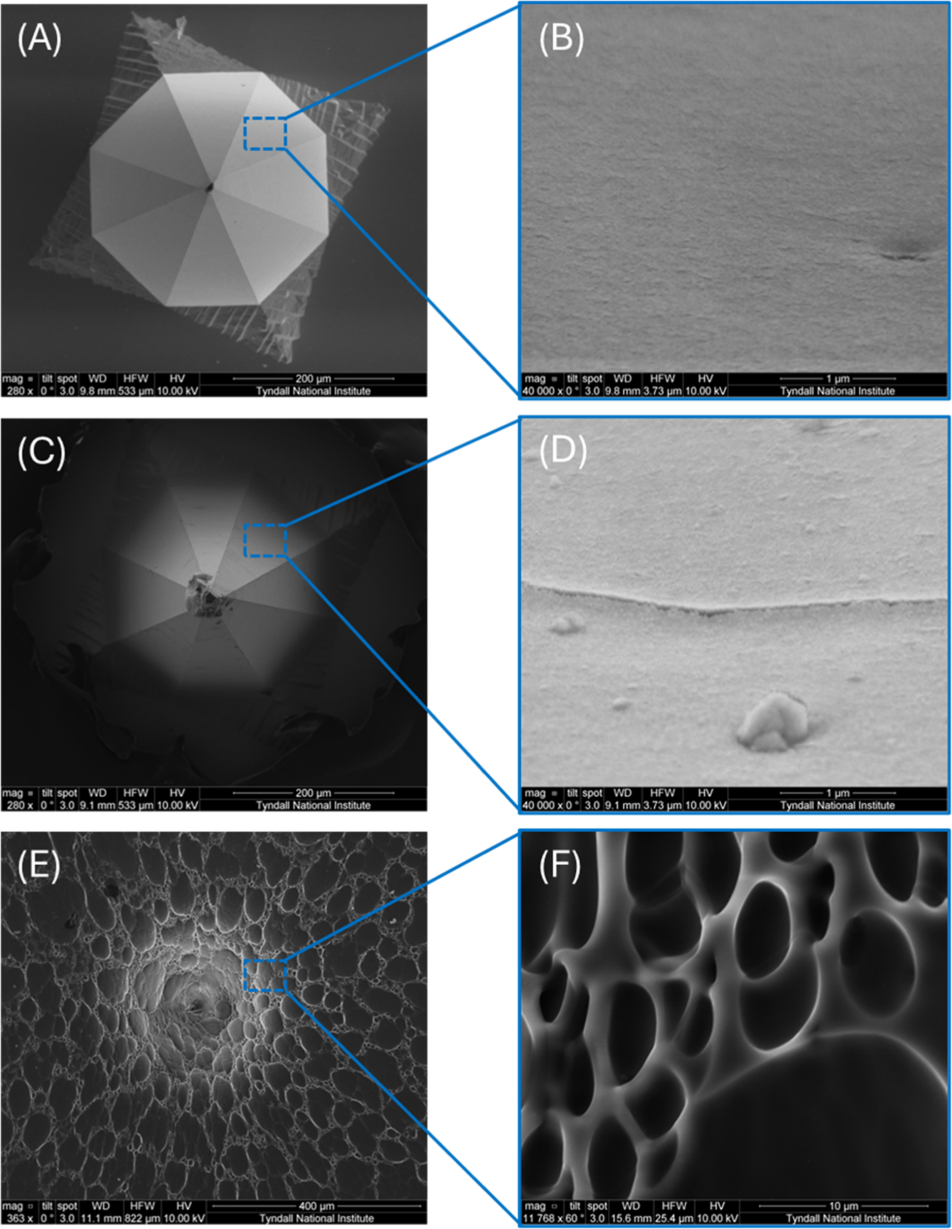
Characterization of the Pt microneedle, PEDOT/Pt microneedle, and ISM/PEDOT/Pt microneedle. (A) SEM image of a Pt microneedle without passivation. (B) Higher-magnification SEM image of the surface of the Pt microneedle. (C) SEM image of a Pt microneedle passivated with ArCare and modified with PEDOT. (D) Higher-magnification SEM image of the surface of a microneedle modified with PEDOT. (E) SEM image of a Pt microneedle passivated with ArCare, modified with PEDOT and coated with an ISM layer. (F) Higher magnification of the surface of a PEDOT/Pt microneedle coated with ISM.

### 3.3 Electrochemical Characterization and Sensing Platform

The first step of the optimization was focused on modification of the microneedle electrodes. The chemical modification of the microneedle array was monitored by observing changes in the electron transfer properties of the microneedle in relation to the electrolyte solution. This characterization was performed by using CV and SWV experiments.

Figure 6 (A) shows a decreased electrical conductivity of the electrode caused by the PEDOT coating, resulting in a decrease in the current responsiveness. The CV profile changes significantly after an overnight application of the ISM, as seen by the blue CV curve in Figure 6 (A) and (D). Changes in the peak-to-peak current difference and potential difference of FcCOOH are noticeable at each step of the modification process. The unaltered microneedle displays a quasi-reversible system with a potential difference (Δ*E*_p_) of 46.39 mV and a current difference (Δ*I*_p_) of 49.5 μA. When PEDOT is electrodeposited on the microneedle array, the surfaces result in a Δ*E*_p_ increase of 17.09 mV and a Δ*I*_p_ reduction of 1 μA. Upon introducing the ISM to the electrode surface, we observe a Δ*E*_p_ reduction by 7.33 mV and a Δ*I*_p_ reduction of 45.9 μA with respect to the K^+^ microneedle sensor. For the CA^2+^ microneedle sensor, we noticed a similar behavior, but the addition of a PEDOT membrane resulted in an increase in conductivity. The system presents a Δ*E*_p_ of 85.5 mV and a Δ*I*_p_ of 42.2 μA for a control microneedle array. When modified with PEDOT, the Δ*E*_p_ remained constant and the Δ*I*_p_ decreased to 6.1 μA. After ISM incubation, the Δ*E*_p_ decreased to 19.53 mV and the Δ*I*_p_ decreased to 33.6 μA.

**Figure 6:**
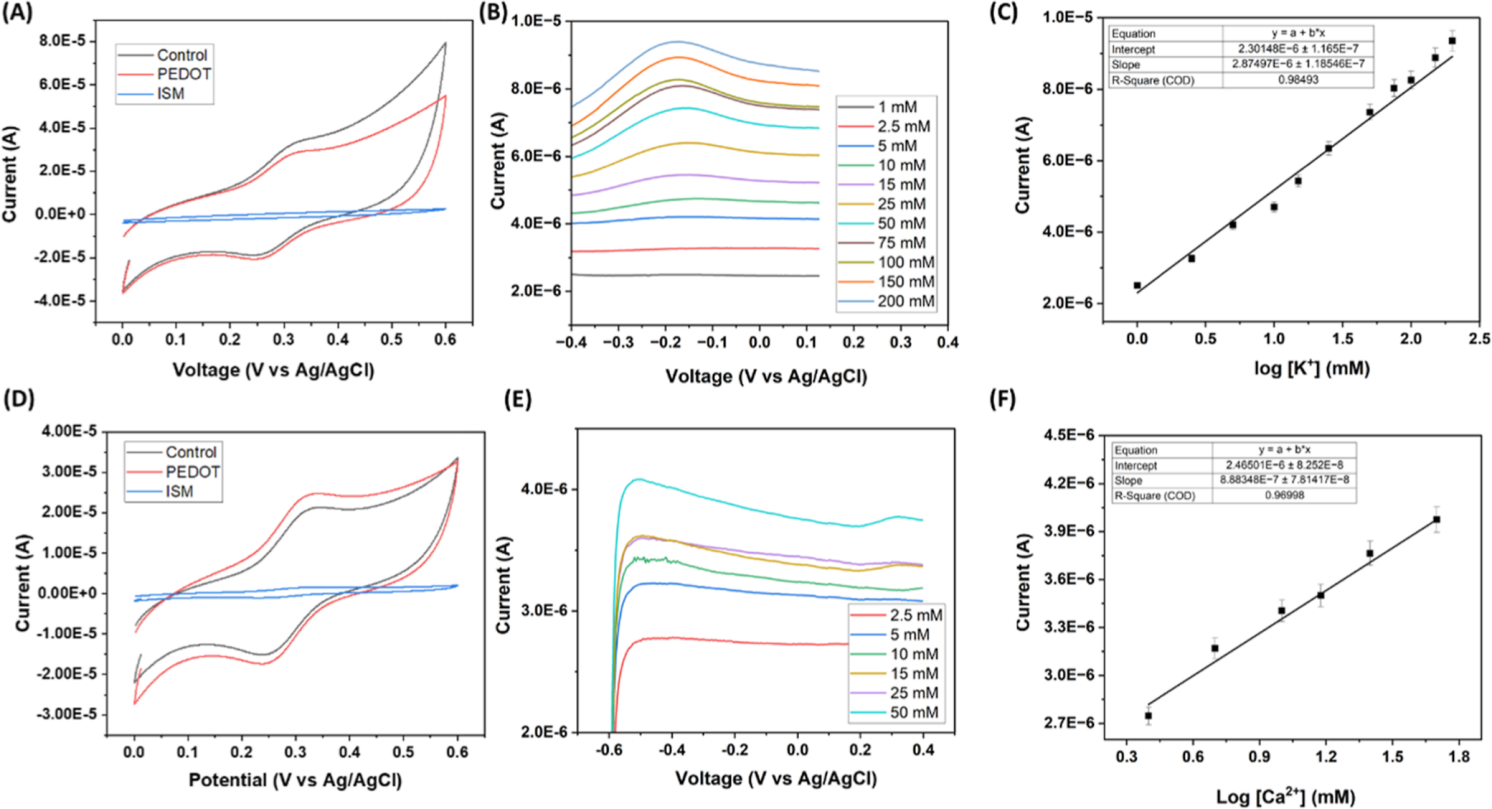
Electrochemical performance of K^+^ and Ca^2+^ ArCare sensors in buffers. (A) Response of a microneedle sensor to different modification stages of a K^+^ sensor. (B) SWV scans of K^+^ concentrations ranging from 1 to 200 mM. (C) Calibration curve shows a linear relationship between the current and the logarithm of K^+^ concentrations (*n* = 6). (D) Response of a microneedle sensor to different modification stages of a Ca^2+^ sensor. (E) SWV scans of Ca^2+^ concentrations ranging from 2.5 to 50 mM. (F) Calibration curve shows a linear relationship between the current and the logarithm of Ca^2+^ concentrations (*n* = 6).

Square wave voltammograms ranging from 1 to 200 mM with a linear increase in current proportional to the concentration can be seen in Figure 6 (B) and (E). Figures 6 (C) and (F) show the calibration curve for the different concentrations of potassium and calcium. The calibration curve was constructed by using peak currents obtained at – 0.2 V from SWV measurements. The final calibration curve is generated by taking the logarithmic values of the dilution concentrations. Both calibration plots confirm a linear increase in current proportional to the concentration with coefficient of determination (*r* ^2^) values of 0.98 and 0.97 for potassium and calcium, respectively.

In their study, Huijie Li et al.^39^ assessed the K^+^ sensor’s performance across a concentration range from 1 mM to 200 mM. Their results demonstrated a consistent and distinct correlation between the concentration and current, suggesting that the sensor is responsive to potassium-level variations and effectively interprets increasing concentrations. The findings of the calibration indicate that the experimental results adhere to this pattern, suggesting that the sensor fabricated with ArCare passivation for K^+^ maintains its sensitivity to varying concentrations.

### 3.4 Selectivity/Interferences of the Microneedle Sensor

The ISF is a complex bodily fluid that contains various components that could potentially interfere with the responses of Ca^2+^ and K^+^ sensors.^40^ To examine the impact of potential interfering compounds on the sensors’ response, this study analyzed the selectivity of Ca^2+^ and K^+^ sensors. Specifically, the current response was recorded upon the addition of physiologically relevant interfering species, KCl, NaCl, CaCl_2_, and MgCl_2_, to ISF. Four separate solutions were prepared, each spiked with one of the above salts, and the same batch of sensors was tested across all solutions; the resulting SWV data were used to generate the graph shown. The results presented in Figure 7 (A,B) demonstrated that the potential signal changes induced by interfering ions are insignificant. Given that the concentration of interfering species was 10-fold higher than their physiological levels in ISF, the results further underscore the stability and specificity of the sensors in complex biological media.

**Figure 7:**
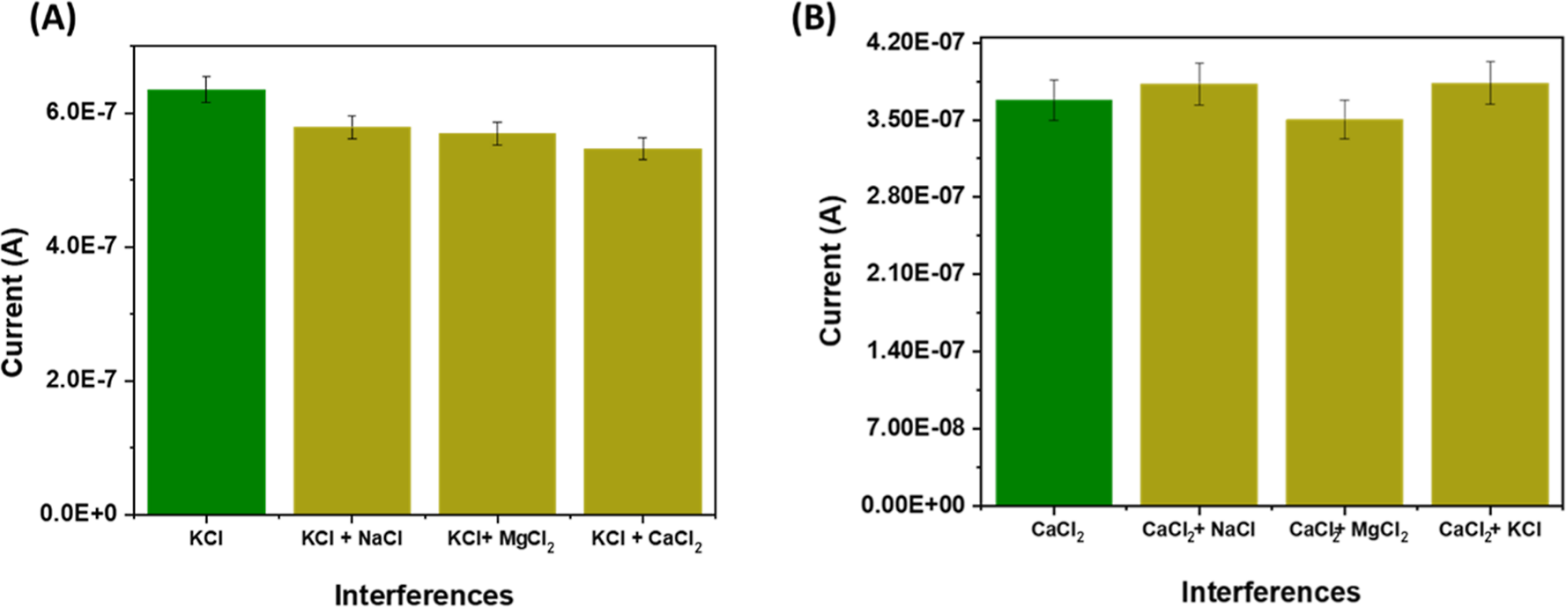
Selectivity/interferences study of K^+^ and Ca^2+^ ArCare sensors. (A) K^+^ sensor in response to different input interferences, including CaCl2, MgCl2, and NaCl. (B) Ca^2+^ sensor in response to different input interferences, including KCl, MgCl2, and NaCl.

These investigations imply that the developed sensors for Ca^2+^ and K^+^ exhibit high selectivity toward various electrolytes commonly found in ISF, facilitating the creation of a multiplexed sensing system. A difference in the response current is observed for K^+^ sensors, which could be due to the variation in the molecular weights of Ca^2+^ and K^+^. The potassium ion, having a lower molecular weight than Ca^2+^, diffuses faster into the ISM, leading to the formation of a K^+^ ionophore complex.

For the K^+^ sensor, when NaCl is introduced as an interfering substance, the current drops from 0.64 μA to 0.58 μA, resulting in an interference of 0.06 μA, i.e., 10.34%. When MgCl_2_ is used as an interfering substance, the current falls to 0.57 μA, which causes an interference of 0.07 μA or 12.28%. When CaCl_2_ is employed, the current reduces to 0.55 μA, causing an interference of 0.09 μA or 16.36% (Table 5). While the highest interference was observed in the presence of calcium, this can be overlooked as the concentration tested is 25 mM and it is 10 times higher than what is found in ISF. Typically, the accepted percentage that can be overlooked is 10%;^41,42^ however, since this test was performed with high concentrations of interfering agents, the sensors functioned exceptionally well. Sensors modified for potassium showed lower interference when tested in sodium and minimal interference for magnesium.

**Table 5:** Ions Interfering for Potassium

ions interfering for potassium	peak current (μA)	interference caused (μA)	percentage interference (%)
potassium (K^+^)	0.64	0	0
potassium (K^+^) with sodium (Na^+^)	0.58	0.06	9.38
potassium (K^+^) with magnesium (Mg^2+^)	0.57	0.07	10.9
potassium (K^+^) with calcium (Ca^2+^)	0.55	0.09	14.1

When NaCl is introduced to the Ca^2+^ sensor as an interfering substance, the current experiences a slight increase from 0.37 μA to 0.38 μA, leading to an interference of 0.01 μA, i.e., 2.63% interference. When MgCl_2_ is used as the interfering substance, the current decreases to 0.35 μA, causing an interference of 0.02 μA or 5.2%. Similarly, the introduction of KCl results in a reduction of the current to 0.38 μA, which corresponds to an interference of 0.01 μA or 2.63% (Table 6). Using the above-mentioned explanation for these sensors, the calcium sensors performed exceptionally well with a high concentration. This shows the reliability of these sensors.

**Table 6:** Ions Interfering for Calcium

ions interfering for calcium	peak current (μA)	interference caused (μA)	percentage interference (%)
calcium (Ca^2+^)	0.37	0	0
calcium (Ca^2+^) with sodium (Na^+^)	0.38	0.01	2.70
calcium (Ca^2+^) with magnesium (Mg^2+^)	0.35	0.02	6.1
calcium (Ca^2+^) with potassium (K^+^)	0.38	0.01	2.70

### 3.5 Detection of K^+^ and Ca^2+^ Microneedle Sensors in Artificial ISF

To assess the performance of the Ca^2+^ sensor, it is assessed in artificial ISF with a physiologically relevant concentration of CaCl_2_ ranging from 1 to 5 mM.^43,44^ The calibration curve is derived by plotting the current against the voltage (Figure 5C). As depicted in Figure 8D, a linear relationship exists between the current and the logarithm of the Ca^2+^ concentration. The sensitivity is 0.02 nM, and the linear regression yields an r^2^ of 0.99 for the Ca^2+^ sensor.

**Figure 8:**
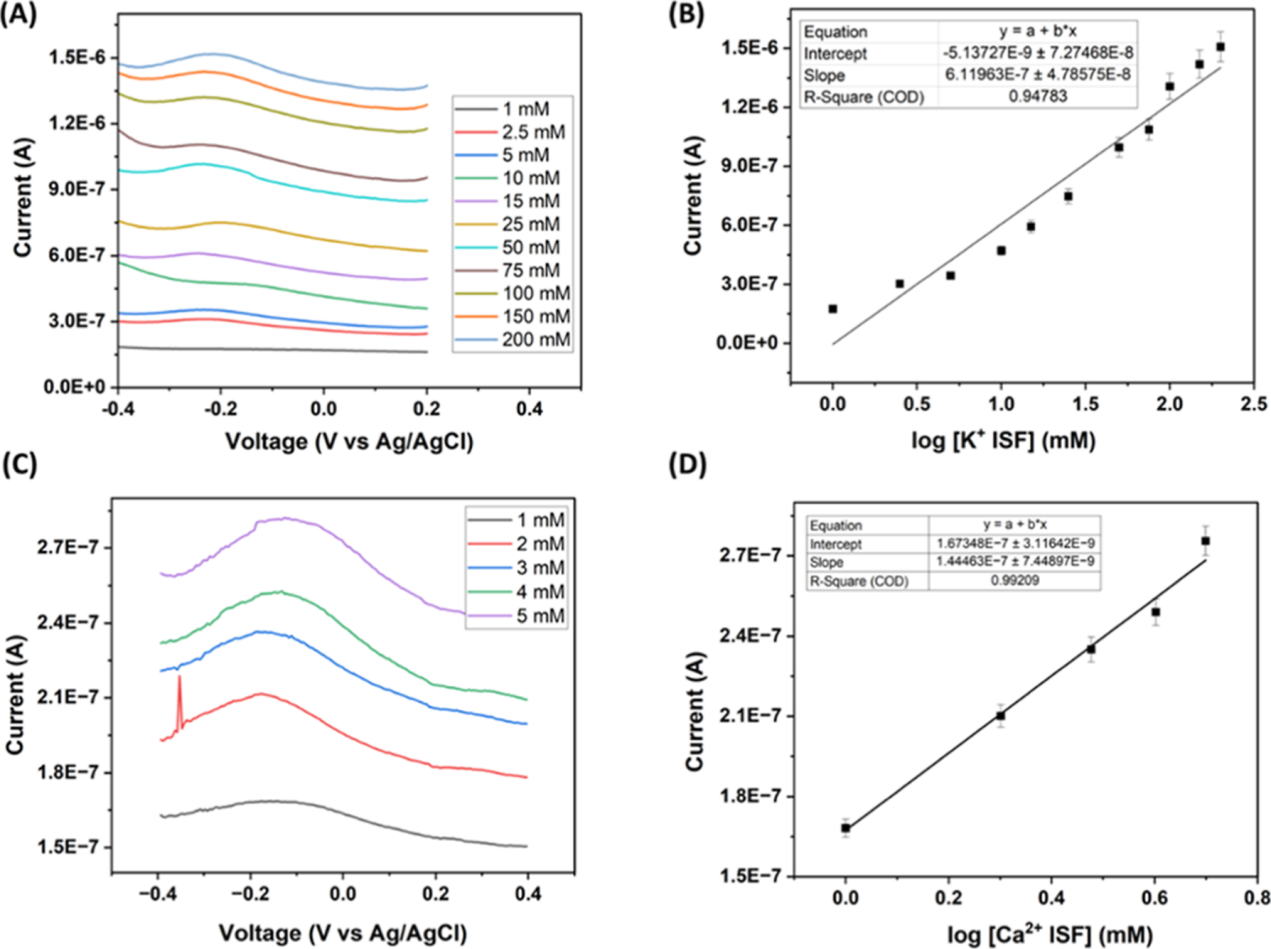
Electrochemical performance of K^+^ and Ca^2+^ ArCare sensors in artificial ISF. (A) SWV scans of K^+^ concentrations in artificial ISF ranging from 1 to 200 mM. (B) Calibration curve shows a linear relationship between the current and the logarithm of K^+^ concentrations in artificial ISF (*n* = 6). (C) SWV scans of Ca^2+^ concentrations in artificial ISF ranging from 1 to 5 mM. (D) Calibration curve shows a linear relationship between the current and the logarithm of Ca^2+^ concentrations in artificial ISF (*n* = 3).

Similarly, the K^+^ sensor is sequentially electrodeposited with PEDOT onto a Pt/ArCare microneedle, followed by drop-casting with an ion-selective membrane. Electrochemical characterization was carried out using varying concentrations of KCl against the background of artificial ISF. Figure 5A illustrates the current increase of the K^+^ sensor corresponding to the rise in KCl concentration within the physiologically relevant range of 1–200 mM in artificial ISF. As depicted in Figure 8B, a linear relationship (*r* ^2^ = 0.95, a sensitivity of 0.099 nM) exists between the current and the logarithm of K^+^ concentration. These electrochemical characterizations validate the ability of the fabricated K^+^ sensor for real-time tracking of K^+^ fluctuations in ISF. The data from the Ca^2+^ and K^+^ sensors indicate that the microneedle sensor displays linear responses to different ion concentration levels within the physiological range of interest.

While the microneedle-based sensor has shown effectiveness in artificial interstitial fluid, applying this technology to real biological samples introduces added complexity. Biological fluids, such as human ISF, plasma, or sweat, contain proteins, lipids, and various metabolites that can interfere with sensor performance through biofouling and matrix effects, potentially reducing sensitivity and selectivity. Protein fouling, in particular, may block ion transport and cause signal drift, but this can be mitigated by using antifouling surface coatings. Additionally, variations in the ionic strength and composition of physiological fluids may require the recalibration of the sensor. The current sensor design aims for robust performance, but further validation with clinical or ex vivo samples is necessary to confirm accuracy, stability, and reproducibility. Despite these challenges, the sensor’s minimally invasive approach and demonstrated selectivity make it a promising tool for real-time, in vivo monitoring, provided further optimization and testing are carried out.

### 3.6 Multiparametric Detection

The performance of the PULSE was benchmarked against that of the benchtop potentiostat to evaluate its capabilities to integrate microneedles in a multiparametric setting. The results of pH detection are shown in Figure 9 (A), demonstrating that the PULSE and the benchtop system exhibit similar trends, with overlapping standard deviations. A correlation analysis of the data sets from both systems produced a coefficient of 0.98, indicating a strong similarity between them. The pH calibration curve showed a slope of 10.24 mV/pH and an *r* ^2^ of 0.96, showing good linearity when using the PULSE.

**Figure 9:**
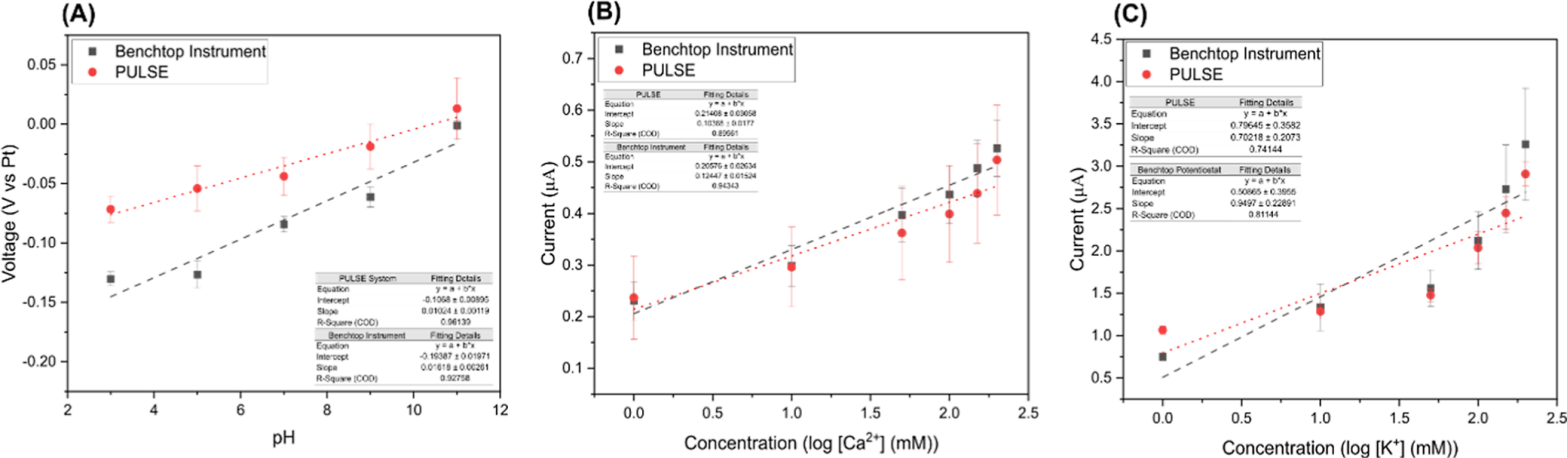
(A) pH calibration curves; (B) Ca^2+^ calibration curve; and (C) K^+^ calibration curves using the benchtop potentiostat and the PULSE. The error bars correspond to the standard error.

The detection of Ca^2+^ and K^+^ is illustrated in Figure 9 (B) and (C), respectively. The results generated by the PULSE platform showed a strong correlation with those from the benchtop system, yielding a coefficient of 0.99 for both the Ca^2+^ and K^+^ measurements. This strong correlation supported the capability of the PULSE to produce comparable results to the benchtop instrument. The PULSE detected K^+^ and Ca^2+^ with a sensitivity of 41.7 nA/mM/mm^2^ and 8.12 nA/mM/mm^2^, respectively. These results indicate the potential for integrating the microneedles with a portable device for use in a variety of applications.

## 4 Conclusion

This study validated microneedle sensors for detecting calcium, potassium, and pH with high selectivity and accuracy. CV and SWV confirmed linear ion concentration responses, while Raman and SEM verified PEDOT and ISM deposition. Surface modifications improved electron transfer and ion specificity, yielding calibration curves with high linearity (*r* ^2^ = 0.98 for potassium and 0.97 for calcium), demonstrating sensor sensitivity. The interference study indicated that potassium sensors experienced some interference, ranging from 9% to 14% depending on the ion. For calcium sensors, interference caused by other ions falls in the range of 2% to 6%. This interference was caused by testing with concentrations that are ten times higher than what can interfere in ISF. Even after extreme conditions, especially calcium sensors performed exceptionally well. The calibrations repeated in the artificial ISF were found to be highly linear (*r* ^2^ = 0.95 for potassium and 0.99 for calcium), thus proving these sensors can effectively detect the desired target in complex media.

Calibration curves obtained with the PULSE showed high linearity and high correlation with the benchtop potentiostat for the detection of pH, Ca^2+^, and K^+^. The calibration r^2^ values obtained using the PULSE setup were 0.96 for pH, 0.94 for calcium, and 0.84 for potassium. These results indicated that this approach is also reliable for multiparametric POC applications and can be further explored in the future as a diagnostic tool. With advantages over existing technologies such as more sensitivity, ease of fabrication, and stability compared to hollow microneedles, this provides a novel approach to the existing technologies.

The current study shows the high selectivity of potassium and calcium detection with passivated microneedle sensors. The successful use of these devices in artificial ISF, along with the compatibility with portable devices, contributes to their potential applicability in diverse fields ranging from real-time health monitoring to nutritional quality control. This work will facilitate progress toward compact, low-cost, and sensitive sensors for use at the POC.

## Supplementary Material



## Notes

Electrochemical performance of K^+^ and Ca^2+^ sensors incorporating NOA68 passivation, results provided for the negative control, electrochemically active surface area across different sensor development layers, repeatability, pH response, and real sample analysis (PDF)

## References

[1] Saputra H. A. (2023). Electrochemical
Sensors: Basic Principles, Engineering, and State of the Art. Monatsh. Chem.

[2] Sharma A., Badea M., Tiwari S., Marty J. L. (2021). Wearable
Biosensors:
An Alternative and Practical Approach in Healthcare and Disease Monitoring. Molecules.

[3] Lubken R. M., de Jong A. M., Prins M. W. J. (2022). Real-Time
Monitoring of Biomolecules:
Dynamic Response Limits of Affinity-Based Sensors. ACS Sens.

[4] Kumar, A. M. ; Kachhawa, K. Biomedical Applications of Bioelectrochemical Sensors; Multifaceted Bio-sensing Technology; Elsevier 2023, pp239–260

[5] Wang Z., Liu N., Ma Z. (2014). Platinum Porous Nanoparticles
Hybrid with Metal Ions
as Probes for Simultaneous Detection of Multiplex Cancer Biomarkers. Biosens. Bioelectron..

[6] Eissa S., Zourob M. (2020). Ultrasensitive Peptide-Based
Multiplexed Electrochemical
Biosensor for the Simultaneous Detection of Listeria Monocytogenes
and Staphylococcus Aureus. Mikrochim. Acta.

[7] Kashefi-Kheyrabadi L., Nguyen H. V., Go A., Baek C., Jang N., Lee J. M., Cho N. H., Min J., Lee M. H. (2022). Rapid,
Multiplexed, and Nucleic Acid Amplification-Free Detection of SARS-CoV-2
RNA Using an Electrochemical Biosensor. Biosens.
Bioelectron..

[8] Yokus M. A., Songkakul T., Pozdin V. A., Bozkurt A., Daniele M. A. (2020). Wearable
Multiplexed Biosensor System toward Continuous Monitoring of Metabolites. Biosens. Bioelectron..

[9] Jia X., Liu Z., Liu N., Ma Z. (2014). A Label-Free Immunosensor Based on
Graphene Nanocomposites for Simultaneous Multiplexed Electrochemical
Determination of Tumor Markers. Biosens. Bioelectron..

[10] Glatz R. T., Ates H. C., Mohsenin H., Weber W., Dincer C. (2022). Designing
Electrochemical Microfluidic Multiplexed Biosensors for On-Site Applications. Anal. Bioanal. Chem..

[11] Liu G. S., Kong Y., Wang Y., Luo Y., Fan X., Xie X., Yang B. R., Wu M. X. (2020). Microneedles for
Transdermal Diagnostics:
Recent Advances and New Horizons. Biomaterials.

[12] Le Z., Yu J., Quek Y. J., Bai B., Li X., Shou Y., Myint B., Xu C., Tay A. (2023). Design Principles of
Microneedles for Drug Delivery and Sampling Applications. Mater. Today.

[13] Zheng L., Zhu D., Xiao Y., Zheng X., Chen P. (2023). Microneedle Coupled
Epidermal Sensor for Multiplexed Electrochemical Detection of Kidney
Disease Biomarkers. Biosens. Bioelectron..

[14] Gao J., Huang W., Chen Z., Yi C., Jiang L. (2019). Simultaneous
Detection of Glucose, Uric Acid and Cholesterol Using Flexible Microneedle
Electrode Array-Based Biosensor and Multi-Channel Portable Electrochemical
Analyzer. Sensors Actuators, B Chem..

[15] Mugo S. M., Robertson S. V., Lu W. (2023). A Molecularly Imprinted Electrochemical
Microneedle Sensor for Multiplexed Metabolites Detection in Human
Sweat. Talanta.

[16] Li H., Wu G., Weng Z., Sun H., Nistala R., Zhang Y. (2021). Microneedle-Based
Potentiometric Sensing System for Continuous Monitoring of Multiple
Electrolytes in Skin Interstitial Fluids. ACS
Sens.

[17] Dervisevic M., Alba M., Yan L., Senel M., Gengenbach T. R., Prieto-Simon B., Voelcker N. H. (2021). Transdermal Electrochemical Monitoring
of Glucose via High-Density Silicon Microneedle Array Patch. Adv. Funct. Mater..

[18] Mishra R. K., Goud K. Y., Li Z., Moonla C., Mohamed M. A., Tehrani F., Teymourian H., Wang J. (2020). Continuous Opioid Monitoring
along with Nerve Agents on a Wearable Microneedle Sensor Array. J. Am. Chem. Soc..

[19] García-Guzmán J. J., Pérez-Ràfols C., Cuartero M., Crespo G. A. (2021). Microneedle
Based Electrochemical (Bio)­Sensing: Towards Decentralized and Continuous
Health Status Monitoring. TrAC - Trends Anal.
Chem..

[20] Jochum F., Moltu S. J., Senterre T., Nomayo A., Goulet O., Iacobelli S., Braegger C., Bronsky J., Cai W., Campoy C., Carnielli V., Darmaun D., Decsi T., Domellöf M., Embleton N., Fewtrell M., Fidler Mis N., Franz A., Goulet O., Hartman C., Hill S., Hojsak I., Iacobelli S., Jochum F., Joosten K., Kolaček S., Koletzko B., Ksiazyk J., Lapillonne A., Lohner S., Mesotten D., Mihályi K., Mihatsch W. A., Mimouni F., Mølgaard C., Moltu S. J., Nomayo A., Picaud J. C., Prell C., Puntis J., Riskin A., Saenz De Pipaon M., Senterre T., Shamir R., Simchowitz V., Szitanyi P., Tabbers M. M., Van Den Akker C. H. B., Van Goudoever J. B., Van Kempen A., Verbruggen S. (2018). ESPGHAN/ESPEN/ESPR/CSPEN Guidelines on Pediatric
Parenteral Nutrition: Fluid and Electrolytes. Clin. Nutr..

[21] McNamara K., Tofail S. A. M. (2017). Nanoparticles in Biomedical Applications. Adv. Phys. X.

[22] Lewis, J. L., III Overview of Calcium’s Role in the Body 2022.

[23] Cleveland clinic , Hypocalcemia.2022

[24] Norman Parathyroid Centre . ’High Blood Calcium, and the Causes of High Calcium in the Blood. 2022.

[25] Xu G., Cheng C., Yuan W., Liu Z., Zhu L., Li X., Lu Y., Chen Z., Liu J., Cui Z., Liu J., Men H., Liu Q. (2019). Smartphone-based battery-free and
flexible electrochemical patch for calcium and chloride ions detections
in bio fluids. Sens. Actuators B-Chem.

[26] Zhao K., Kang B., Zhou B. (2022). Wearable electrochemical
sensors
for monitoring of inorganic ions and pH in sweat. Int. J. Electrochem. Sci..

[27] Huang X., Zheng S., Liang B., He M., Wu F., Yang J., Chen H.-J., Xie X. (2023). 3D-assembled
microneedle
ion sensor-based wearable system for the transdermal monitoring of
physiological ion fluctuations. Microsyst Nanoeng.

[28] McConville A., Davis J. (2016). Electrochem commun, Transdermal microneedle
sensor arrays based on
palladium. Polym. Compos..

[29] Neville M. C., Watters C. D. (1983). J Dairy Sci, Secretion of Calcium
into Milk. Review.

[30] Takeuchi K., Takama N., Sharma K., Paul O., Ruther P., Suga T., Kim B. (2022). Microfluidic
chip connected to porous
microneedle array for continuous ISF sampling. Drug Deliv Transl Res..

[31] Cheng Y., Luan X., Weng J., Zhang L., Ye F. (2024). Engineering
sampling microneedles for biomolecules sensing. Chem. Eng. J..

[32] Lu H., Zada S., Yang L., Dong H. (2022). Microneedle-Based Device
for Biological Analysis. Front. Bioeng. Biotechnol.

[33] Bocchino, A. ; Teixeira, S. R. ; Iadanza, S. ; Melnik, E. ; Kurzhals, S. ; Mutinati, G. C. ; O’Mahony, C. Development and Characterization of Passivation Methods for Microneedle-based Biosensors, 2022 44th Annual International Conference of the IEEE Engineering in Medicine & Biology Society (EMBC), IEEE, Glasgow, Scotland, United Kingdom, 2022; pp 1275–1278 10.1109/EMBC48229.2022.9871005 36086365

[34] Ferreira C., Barry F., Todorović M., Sugrue P., Teixeira S. R., Galvin P. (2025). PULSE: A Fast Portable Unit for Lab-on-Site Electrochemistry. Sensors.

[35] Bretag A. H. (1969). Synthetic
interstial fluid for isolated mammalian tissue. Life Sci..

[36] Sakmeche N., Aeiyach S., Aaron J. J., Jouini M., Lacroix J. C., Lacaze P.-C. (1999). Improvement of the
electrosynthesis and physicochemical
properties of poly­(3,4-ethylenedioxythiophene) using a sodium dodecyl
sulfate micellar aqueous medium. Langmuir.

[37] Tamburri E., Orlanducci S., Toschi F., Terranova M. L., Passeri D. (2009). Growth mechanisms, morphology, and electroactivity
of PEDOT layers produced by electrochemical routes in aqueous medium. Synth. Met..

[38] Du X. S., Yu Z. Z., Dasari A., Ma J., Mo M., Meng Y., Mai Y. W. (2008). New method to prepare
graphite nanocomposites. Chem. Mater..

[39] Li H., Wu G., Weng Z., Sun H., Nistala R., Zhang Y. (2021). Microneedle-Based
Potentiometric Sensing System for Continuous Monitoring of Multiple
Electrolytes in Skin Interstitial Fluids. ACS
Sens.

[40] Samant P. P., Niedzwiecki M. M., Raviele N., Tran V., Mena-Lapaix J., Walker D. I., Felner E. I., Jones D. P., Miller G. W., Prausnitz M. R. (2020). Sampling interstitial fluid from human skin using amicroneedle
patch. Sci. Transl. Med..

[41] Li Y., Luo Z., Li G., Belwal T., Li L., Xu Y., Su B., Lin X. (2021). Interference-free detection of caffeine in complex
matrices using a nanochannel electrode modified with binary hydrophilic–
hydrophobic pdms. ACS Sens.

[42] Westgard, J. O. ; Barry, P. L. ; Quam, E. F. Basic method validation: training in analytical quality management for healthcare laboratories; Westgard Quality Corporation, 1999.

[43] Heikenfeld J., Jajack A., Feldman B., Granger S. W., Gaitonde S., Begtrup G., Katchman B. A. (2019). Accessing
analytes inbiofluids for
peripheral biochemical monitoring. Nat. Biotechnol..

[44] Dervisevic M., Alba M., Prieto-Simon B., Voelcker N. H. (2020). Skin in the diagnostics
game: Wearable biosensor nano- and microsystems for medical diagnostics. Nano Today.

