# Supplementary material for: A Microneedle-Based Electrochemical Sensor with Multiplex Detection in Physiological Environment and Incorporated in a Portable Device
Source: ACS Omega. 2025 Aug 5;10(32):36203–15. doi: 10.1021/acsomega.5c04024 (PMC12368728; doi:10.1021/acsomega.5c04024)
Supplement: Supplementary file 1 [file ao5c04024_si_001.pdf]

Supplementary information

Microneedle based electrochemical sensor with multiplex detection in physiological environment and incorporated in a portable device

Chinmay Thatte[1], Fiona Barry[1], Cláudia Ferreira[1], Eoin O'Keeffe[1,2], Valeria Cioce[1,3], Paul Galvin[1], Sofia Rodrigues Teixeira[1]

[1] Tyndall National Institute, University College Cork, Lee Malting, Cork Ireland. Ireland.

[2] School of Biological Sciences, Munster Technological University, Ireland.

[3]School of Biomedical Engineering, Politecnico di Torino, Italy.

Correspondence address: Tyndall National Institute, Lee Maltings, University College Cork, Cork, T12 R5CP Ireland. E-mail address: chinmay.thatte@tyndall.ie

The data presented here pertains to the electrochemical performance of K^+ and Ca^{2+} sensors with NOA68 passivation. Additionally, we include the negative control, electrochemical active area for various sensor development layers, repeatability, pH response, and real sample testing.

NOA68 as Passivation

A spin coater model WS-400BZ-6NPP/LITE (Laurell Technologies Corporation, USA) was used to cover the microneedle arrays with a layer of NOA68 (Norland Optical Adhesive, USA). MN arrays were placed onto a silicon wafer and put in the machine. First step, run for 5 seconds at 500 rpm, second step, 10 seconds at 1000 rpm, and the third step, for 60 seconds at 2500 rpm. Afterwards, the arrays were cured in a UV oven for 60s.

NOA68 is particularly advantageous in studies where interference from the conductive microneedle surface needs to be minimized. The lower current levels facilitate the isolation and quantification of the microneedle's specific electrical characteristics or responses, without significant influence from other factors. The findings of this paper were obtained using ArCare for passivation, however, details about a developed sensor for K^+ using NOA68 as passivation can be found in the supplementary information.

Electrochemical performance for K^+ sensors

Changes in the peak-to-peak current difference and potential difference of the FcCOOH are evident at each stage of the modification process. The unmodified microneedle exhibits a quasi-reversible system with a ΔEp of 100.1mV and a ΔIp of 0.149μA. When PEDOT is electrodeposited on the microneedle array, the surfaces lead to a decrease in ΔEp by 9.77mV and a decrease in ΔIp of 0.11μA. When the

ISM is introduced to the electrode surface, we see an increase in ΔEp by 7.32mV and a decrease in ΔIp of 0.0088µA.

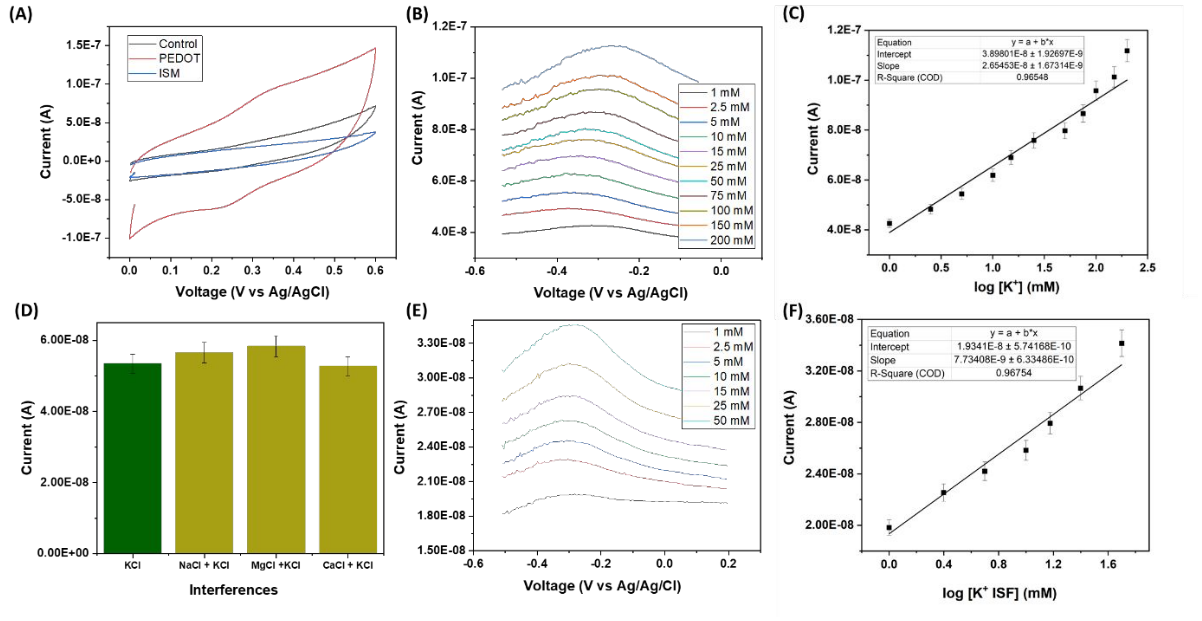

Figure S1: Electrochemical performance of K^+ NOA68 sensors. (A) Response of a microneedle *sensor to different modifications stages of a K^+ sensor. (B) SWV scans of K^+ concentrations ranging from 1 to 200mM. (C) Calibration curve shows a linear relationship between the current and the logarithm of K^+ concentrations (n = 6). (D) K^+ sensor in response to different input interferences, including $CaCl_2$, $MgCl_2$, and NaCl. (E) SWV scans of K^+ concentrations in artificial ISF ranging from 1 to 50mM. (F) Calibration curve shows a linear relationship between the current and the logarithm of K^+ concentrations in artificial ISF (n = 6).*

For the K^+ sensor, when NaCl is introduced as an interfering substance, the current increases from 0.54µA to 0.57µA, resulting in an interference of 0.03µA. When $MgCl_2$ is used as an interfering substance, the current increases to 0.58µA, with an interference of 0.04µA. When $CaCl_2$ is employed, the current reduces to 0.53µA, causing an interference of 0.01µA.

Electrochemical performance for Ca^{2+} sensors

The unmodified microneedle exhibits a quasi-reversible system with a ΔEp of 75.68mV and a ΔIp of 3.9µA. When PEDOT is electrodeposited on the microneedle array, the surfaces do not change for ΔEp but have an increase in ΔIp of 0.69µA. When the ISM is introduced to the electrode surface, we see an increase in ΔEp by 7.32mV and an increase in ΔIp of 3.93µA.

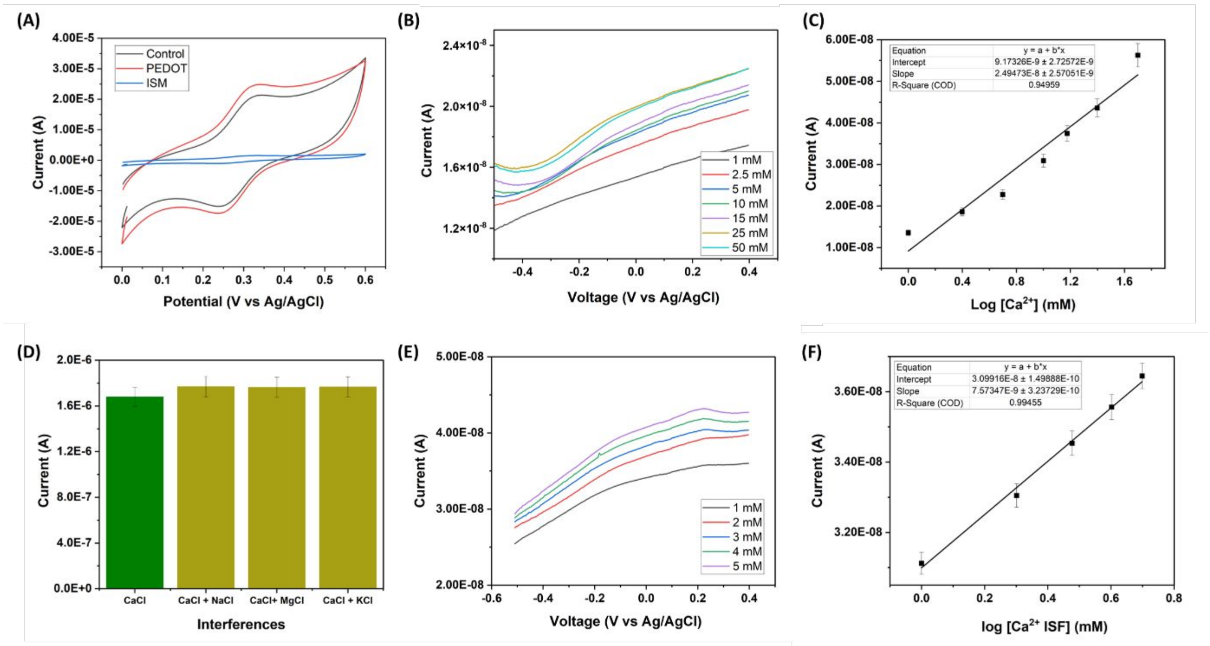

Figure S2: Electrochemical performance of Ca^{2+} NOA68 sensors. (A) Response of a microneedle sensor to different modifications stages of a Ca^{2+} sensor. (B) SWV scans of Ca^{2+} concentrations ranging from 1 to 50mM. (C) Calibration curve shows a linear relationship between the current and the logarithm of Ca^{2+} concentrations (n = 6). (D) Ca^{2+} sensor in response to different input interferences, including KCl, $MgCl_2$, and NaCl. (E) SWV scans of Ca^{2+} concentrations in artificial ISF ranging from 1 to 50mM. (F) Calibration curve shows a linear relationship between the current and the logarithm of Ca^{2+} concentrations in artificial ISF (n = 6).

The sensitivity is 0.0075nM, and the linear correlation coefficient (r^2) is 0.99 for the Ca^{2+} sensor. This indicates that the fabricated Ca^{2+} sensor has the capability to detect Ca^{2+} quantitatively in artificial ISF.

Negative controls

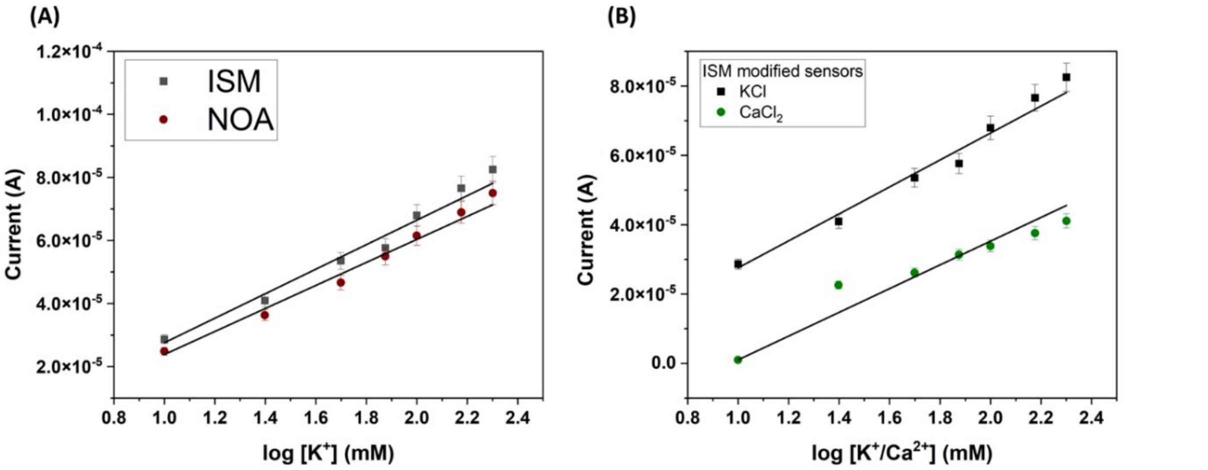

Figure S3: Negative controls electrochemical performance of K^{+} and Ca^{2+} NOA68 sensors. (A) Calibration curves show a linear relationship between the current and the logarithm of K^{+} concentrations in response of a microneedle sensor using the ISM as the base (black line) and NOA68 as the sensor (blue line) (n=6) (B) Calibration curve shows a linear relationship between the current and the logarithm of K^{+} and Ca^{2+} concentrations (n = 6) using an ISM potassium sensor to detect CA^{2+}.

Electrochemical active area

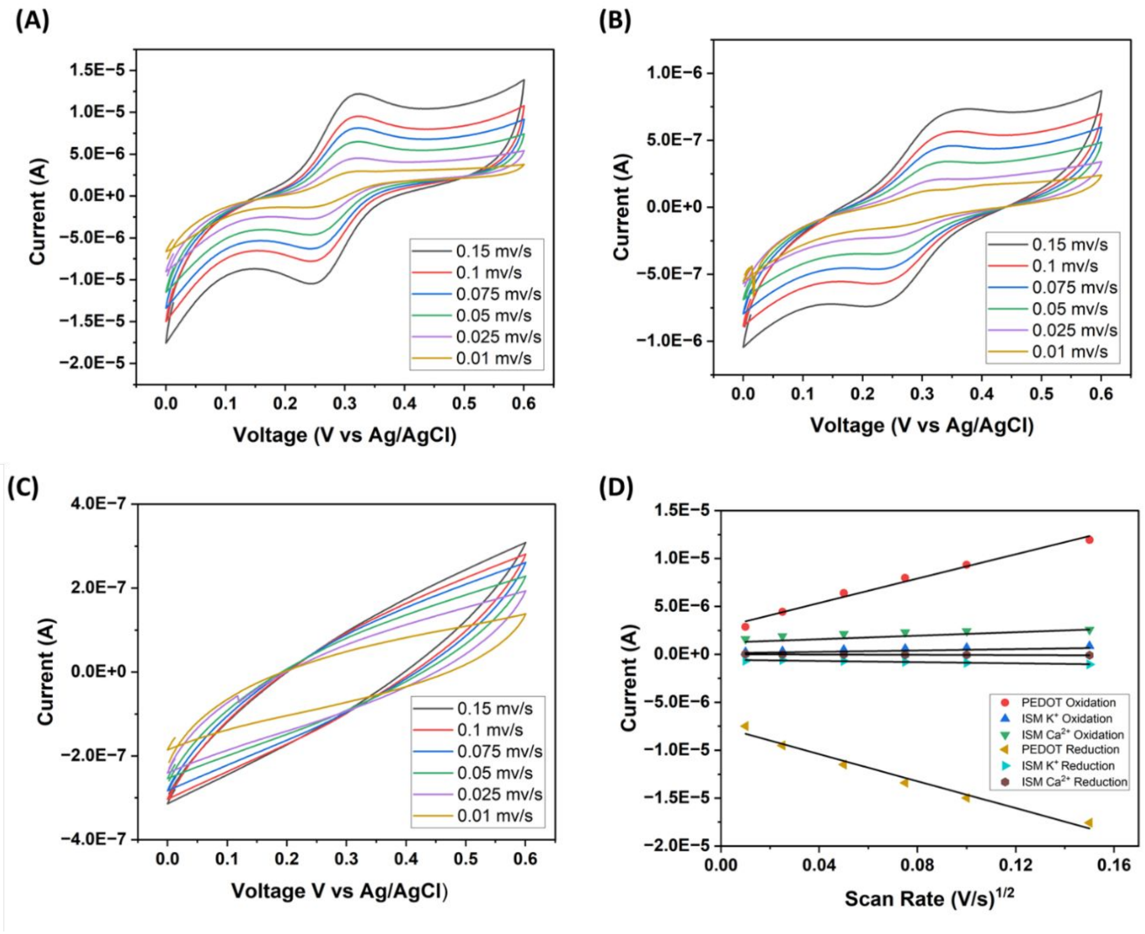

Figure S4: CVs measured on Pt microneedle electrode at increasing scan rates (10 mV/s up to 150 mV/s). (A) PEDOT membrane. (B) Ion Selective membrane for K^+. (C) Ion Selective membrane for Ca^{2+}. (D) Corresponding plot of peak current vs. $v^{1/2}$ for the different layers of the sensor.

Repeatability performance

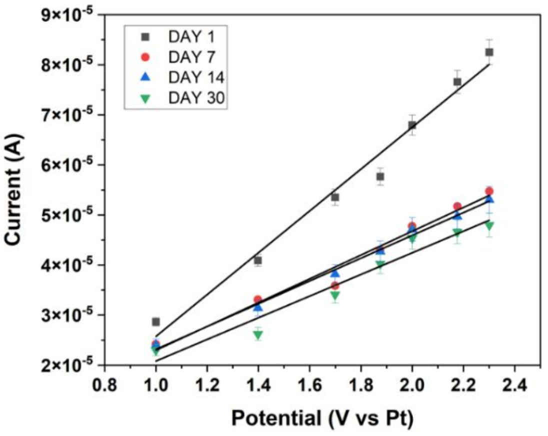

Figure S5: Repeatability/Life shelf of the electrochemical performance of K^+ NOA68 sensors.

Electrochemical performance of pH NOA68 sensors

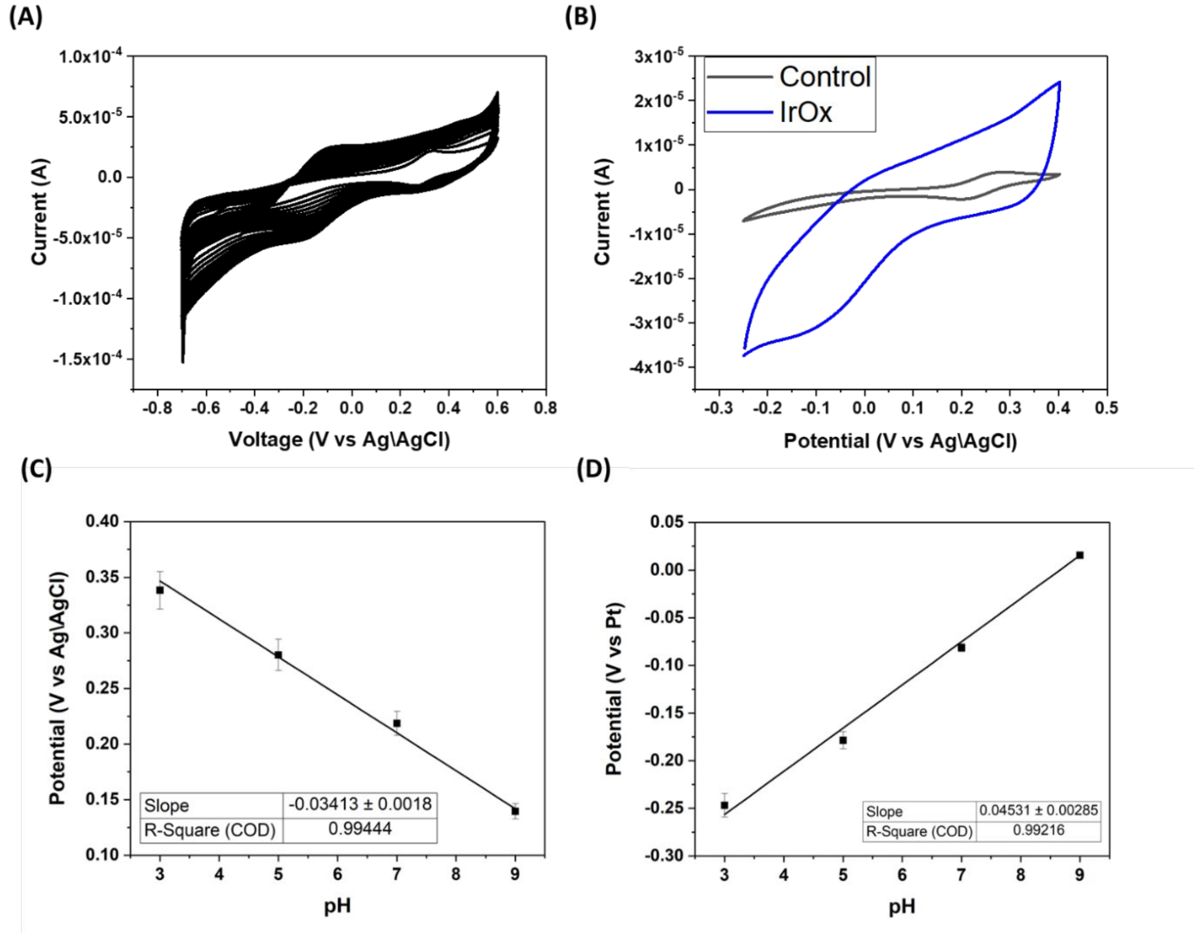

Figure S6: Electrochemical performance of pH NOA68 sensors. (A) Synthesis of IrOx on the microneedle NOA68 sensor (60 cycles were performed with a scan rate of 50 mV/s); (B) CV records

after modification of IrOx- microneedle *NOA68 sensor. (C) Calibration curve shows a linear relationship between the potential and the pH ranging from 3 to 9 using an external RE (Ag/AgCl). (D) Calibration curve shows a linear relationship between the potential and the pH ranging from 3 to 9 using an internal RE (unpassivated Pt microneedle).*
